# Current strategies for prevention of cancer therapy-related cardiotoxicity: pharmacological, non-pharmacological and emerging approaches

**DOI:** 10.3389/fcvm.2025.1668308

**Published:** 2025-10-23

**Authors:** Michele Migliari, Luca Fazzini, Nicola Campana, Martino Deidda, Mariele Dessì, Christian Cadeddu Dessalvi

**Affiliations:** Department of Medical Sciences and Public Health, University of Cagliari, Cagliari, Italy

**Keywords:** cardio-oncology, prevention, cardiotoxicity, cancer therapy-related cardiac dysfunction, pharmacological strategies, non-pharmacological strategies, artificial intelligente

## Abstract

**Background:**

Cardiotoxicity is a major concern in cancer survivors, potentially compromising treatment efficacy, quality of life and long-term survival. With increasing survival rates, the need for effective cardioprotective strategies has become paramount.

**Objective:**

This narrative review evaluates current pharmacological, non-pharmacological, and emerging strategies for preventing cancer therapy-related cardiac dysfunction (CTR-CD), emphasizing recent advances, their clinical applicability and research gaps.

**Methods:**

We conducted a narrative review based on a non-systematic search of PubMed/MEDLINE, Scopus, and Web of Science up to June 2025, focusing on clinical trials, meta-analyses, guideline recommendations, and key observational studies relevant to CTR-CD prevention.

**Results:**

Among pharmacological approaches, renin-angiotensin-aldosterone system inhibitors (RAASi) and beta-blockers modestly preserve left ventricular ejection fraction (LVEF), though benefits on hard outcomes remain unproven. Dexrazoxane is the only FDA-approved agent and shows robust protection in anthracycline-treated patients. Statins and metformin demonstrate promising but still investigational cardioprotective effects, while sodium-glucose cotransporter-2 inhibitors (SGLT2i) show encouraging pilot data. Non-pharmacological strategies—including structured exercise, mediterranean diet, nutritional support and aggressive control of risk factors—are guideline-endorsed, although most evidence relies on surrogate endpoints. Emerging tools such as telemedicine, artificial intelligence and omics sciences offer innovative opportunities for personalized prevention but require multicenter validation.

**Conclusion:**

An integrated, multidisciplinary approach combining both pharmacological and non-pharmacological strategies is essential to effectively prevent cardiotoxicity in cancer patients. Current evidence supports dexrazoxane, risk factor control and selective use of RAASi or beta-blocker in high-risk patients. Exercise and nutrition provide functional and quality of life benefits, while several novel strategies remain exploratory. Future large-scale, multicenter, randomized trial are needed to harmonize international guidelines and define the most effective, sustainable prevention models across diverse patient populations.

## 1 Introduction

In recent years, cancer incidence has shown variable trends depending on tumour site. A stable or slightly increasing trend has been observed for breast and prostate cancers, while a significant reduction has been recorded for lung cancer, largely due to prevention policies and anti-smoking campaigns (1). In Europe, epidemiological data partially reflect trends reported in American registries, with more pronounced increases observed in cancers associated with obesity and smoking (2). In the United States, the cancer mortality rate has steadily declined since 1991, reaching an overall reduction of up to 31% by 2018 (1). Similarly, in Europe, cancer-related mortality is generally decreasing thanks to effective prevention strategies, early diagnosis, and increasingly innovative treatments (2). This progress has led to a significant increase in 5-year survival rates (3), resulting in a growing population of cancer survivors (4). However, the survival benefits are offset by the risk of long-term toxicities, particularly cardiovascular toxicity, which represents one of the most serious complications of oncological treatments (5, 6).

**Figure 1:**
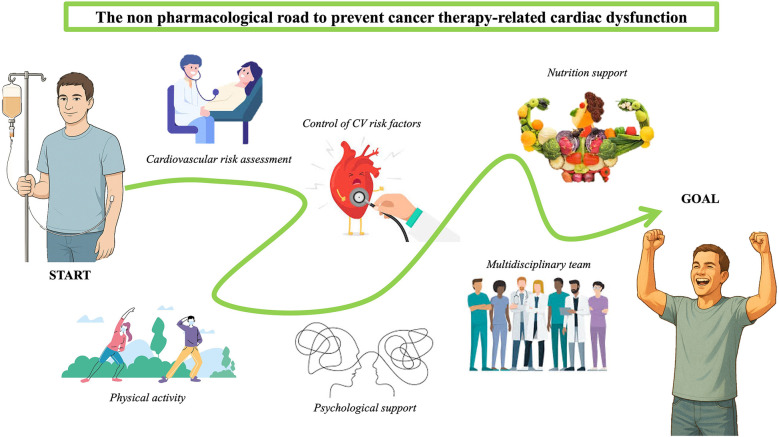
Non-pharmacological strategies to prevent CTR-CD, a schematic image.

Cardiovascular toxicity can significantly affect patients’ quality of life and, in some cases, become a leading cause of morbidity and mortality, especially in the elderly population (7, 8). This complex interaction has underscored the need for shared European cardio-oncology guidelines that encourage an integrated approach between oncologists and cardiologists in managing cardiovascular risk in cancer patients, to prevent and mitigate treatment-related toxicities (9). To this end, initial comprehensive risk assessment—including cardiovascular risk stratification—of cancer patients is crucial (10), along with monitoring during therapy to detect early signs of cardiovascular toxicity and implementing prompt pharmacological and non-pharmacological strategies (9, 10).

The aim of this review is to examine the current state of the art of both pharmacological and non-pharmacological strategies for the prevention of chemotherapy-induced cardiotoxicity, with particular emphasis on the most recent evidence.

## 2 Chemotherapy-related cardiac dysfunction

Chemotherapy-related cardiac dysfunction (CTR-CD) is one of the most feared complications of cancer therapy, with an estimated incidence of up to 10% in cancer patients (11). However, this figure may be underestimated, as subclinical forms detectable only via biomarkers or advanced imaging techniques may also be present. CTR-CD may occur during or after cancer therapy and can negatively impact treatment efficacy, quality of life, and overall survival (12–14) This dysfunction is heterogeneous, resulting from both direct (dose-dependent or dose-independent) and indirect mechanisms, and may be either reversible or permanent (11, 15). Although the pathogenesis is not yet fully elucidated, proposed mechanisms include oxidative stress, mitochondrial dysfunction, and irreversible cardiomyocyte damage (15, 16). Recent evidence also suggests a protective role of the endothelium in preserving myocardial integrity (17).

European guidelines have proposed a universal definition of CTR-CD, distinguishing between symptomatic and asymptomatic forms (18). Each form can present with varying degrees of severity: mild, moderate, or severe (very severe in the case of symptomatic CTR-CD) (13). Symptomatic forms are characterised by signs and symptoms of heart failure, while asymptomatic (subclinical) forms are detected using laboratory tests (troponin, BNP/NT-proBNP) or advanced imaging (echocardiography or cardiac magnetic resonance). Early identification of subclinical toxicity is of paramount importance, as it is a significant predictor of subsequent overt cardiac dysfunction, allowing for a reduction in the risk of progression to more severe clinical forms and improving both quality of life and survival (12, 19). Timely detection enables treatment protocols to be adapted or modified, avoiding unnecessary interruptions and optimizing antitumour efficacy (15).

Accordingly, the use of sensitive diagnostic tools capable of identifying functional and structural changes before clinical manifestations occur is essential. Biomarkers (troponin and BNP) facilitate early detection of myocardial injury (15).

Advanced imaging techniques such as speckle-tracking echocardiography (global longitudinal strain or GLS) and cardiac magnetic resonance (CMR) with parametric mapping can detect structural and functional changes before a significant drop in ejection fraction is observed (20, 21). Indeed, the biplane 2D ejection fraction method has several significant limitations: its reduction is only evident in advanced stages of dysfunction, when the damage may already be substantial and potentially irreversible; it is highly operator-dependent and subject to the variable loading conditions seen in oncology patients due to therapy and its gastrointestinal side effects (22). GLS helps overcome some of these limitations, although it remains sensitive to blood pressure and afterload variations, which may lead to false positives or negatives (23, 24). Myocardial work (MW), which incorporates systolic blood pressure, reduces this dependency and provides a more accurate assessment of myocardial function. Several studies, although with conflicting results, have shown that MW indices [Global Work Index [GWI], Global Constructive Work [GCW], and Global Work Efficiency [GWE]] change early during chemotherapy and may help differentiate between true myocardial dysfunction and blood pressure-related variations (23, 24). The combined use of MW and GLS, rather than their isolated application, improves diagnostic accuracy compared to the use of traditional parameters alone (24).

Finally, three-dimensional ejection fraction (3D-EF) reduces inter-operator variability and improves the diagnostic accuracy of this method; however, recent studies suggest it is less sensitive than GLS in detecting early subclinical dysfunction (25, 26). The implementation of monitoring strategies based on early detection of subclinical damage—through regular surveillance during and after cancer therapy—therefore represents a key element for timely intervention with pharmacological and non-pharmacological strategies to protect the hearts of cancer patients (12).

## 3 Pharmacological strategies

### 3.1 Renin-angiotensin-aldosterone system inhibitors (RAASi)

Renin-angiotensin-aldosterone system inhibitors (RAASi) have been widely studied for their potential cardioprotective effects in patient undergoing cancer therapy. The strongest evidence concerns anthracycline-induced cardiotoxicity, while data for other setting (e.g., trastuzumab, immune checkpoint inhibitors) are more limited. The biological rationale is strong, given their role in counteracting oxidative stress, fibrosis and ventricular remodelling. Clinical trials have tested angiotensin-converting enzyme inhibitors (ACEi), angiotensin receptor blockers (ARBs) and mineralocorticoid receptor antagonists (MRAs), with varying degrees of success (27, 28).

Given the heterogeneity and number of available studies, the main randomized and controlled trials are summarized in Table 1.

**Table 1:** Key clinical trial of RAASi inhibitors in the prevention of chemotherapy-related cardiac dysfunction.

Study (year)	Population	Intervention	Primary endpoint	Main results	Clinical implications
Cardinale (2006)	114 pts, anthracyclines	Enalapril vs. control	Systolic dysfunction	Complete prevention of LVEF decline with enalapril	Proof-of-concept for early ACEi therapy
ICOS-ONE (2018)	273 pts, anthracyclines	Enalapril “troponine-guided” vs. prophylactic	LVEF decline	Both strategies effective; troponin-guided more practical	Supports biomarker-driven approach
OVERCOME (2013)	90 pts, hematologic cancers	Enalapril + Carvedilol vs. placebo	LVEF + MACE	Preserved LVEF, fewer major events	Combination therapy appears protective
PROACT (2025)	400 pts, high-dose anthracyclines	Enalapril + SOC vs. SOC	CTR-CD	No significant difference	Questions routine prophylactic use
SAFE (2025)	190 pts, breast cancer	Ramipril vs. placebo	LVEF + troponin	Attenuated LVEF decline and subclinical injury	Suggests ramipril as an alternative option
PRADA (2016)	120 pts, early breast cancer	Candesartan +/- Metoprolol	LVEF (MRI)	Candesartan attenuated LVEF decline; metoprolol neutral	Robust support for ARBs use
Akpek (2015)	83 pts, anthracyclines	Spironolactone vs. control	LVEF + diastolic function	Preserved systolic and diastolic function	MRAs promising in small RCTs
Meta-analyses (2015–2025)	500–1,000 pts across RCTs	ACEi/ARB +/- MRAs	LVEF, HF events	Consistent modest preservation of LVEF; no reduction in HF incidence	Benefit limited to surrogate outcomes

SOC, standard of care; LVEF, left ventricular ejection fraction; RAASi, rening-angiotensin-aldosterone system inhibitors; ACEi, angiotensin-converting enzyme inhibitors; ARBs, angiontensin II receptor blockers; MRAs, mineralocorticoid receptor antagonists; MRI, magnetic resonance imaging; RCT, randomized clinical trials; MACE, major adverse cardiac events; CTR-CD, cancer therapy related cardiac dysfunction.

**Table 2:** HFA-ICOS score.

Component		Definition	Score
H	History	Prior history of cardiovascular disease (e.g., CAD, HF, arrhythmia)	3
F	Function	Abnormal cardiac function (e.g., reduced LVEF, diastolic dysfunction, LVH)	2–3
A	Biomarkers (assessment)	Elevated	2–3
I	imaging	Abnormal findings on baseline imaging	2
C	Co-morbidities	Hypertension, dyslipidemia, diabetes, renal dysfunction, etc	1–2
O	Oncologic therapy-related risk	Type of cancer therapy (e.g., anthracyclines, HER2 inhibitors, radiation, etc)	1–3
S	Stage or summary	Total score determines risk category: low, moderate, high or very high	0–3 = Low4–6 = Moderate 7–9 = High ≥ 10 = Very High

CAD, coronary artery disease; HF, heart failure; LVEF, left ventricular ejection fraction; LVH, left ventricular hypertrophy.

**Table 3:** A schematic visualization of different type of physical activity in cardio-oncology.

Key features	Aerobic Training	Resistance Training
Main goal	Improve cardiovascular health, VO2-peak and functional capacity	Preserve/increase muscle mass and strength; counteract fatigue and sarcopenia
Initial assessment	Cardiovascular and cancer therapy-related cardiotoxicity risk stratification	Estime maximal strength (with maximum repetition RM or via Brzycki formula)
Training type	Moderate-intensity continuous training (MICT) or High-intensity interval training (HIIT)	Strength, Hypertrophy or Endurance
Recommended frequency	3–5 days/week	2–4 days/week depending on goal (strength, hypertrophy or endurance)
Session duration	30–60 min	Varies with reps; 30–45 min/session
Total weekly time	- Moderate: 150–300 min/week - Vigorous: 75–150 min/week	Dependent on program; minimum 2–4 sessions/week
Precautions	Avoid overexertion if impaired cardiac function	Start with low loads in frail patients
Expected benefits	↑ VO₂peak, ↓ cardiovascular risk, ↑ functional capacity, ↓ fatigue, ↑ survival	↑ strength and lean mass, ↓ sarcopenia, ↑ metabolic health, ↑ treatment tolerance

Overall, ACE inhibitors, particularly enalapril, have been the most studied. Early RCTs such as Cardinale (2006), ICOS-ONE (2018) and OVERCOME (2013) suggested that enalapril could effectively prevent LVEF decline and, in combination with carvedilol, reduce major cardiac events. However, the more recent PROACT trial (2025), the largest to date, failed to demonstrate benefit in high-risk patients on high-dose anthracyclines, thus tempering enthusiasm for routine use. The SAFE trial (2025) with ramipril provided additional signals of benefit in breast cancer patients, though further validation is required (29–33).

Evidence for ARBs is less robust but supportive. The PRADA trial (2016) demonstrated that candesartan attenuated LVEF decline assessed with cardiac MRI (magnetic resonance imaging), while smaller studies with telmisartan reported preservation of diastolic function (34, 35).

MRAs, particularly spironolactone, also showed preservation of systolic and diastolic function in small trials and appear highly effective in network meta-analyses, though large-scale confirmation is lacking (36).

Given the variability across individual trials, meta-analyses offer a more consistent and comprehensive assessment of the cardioprotective effects of RAAS inhibitors in patients undergoing anthracycline-based chemotherapy. Overall, these analyses (Yun 2015, Dong 2020, Caspani 2021, Harmouch 2025) consistently indicated that RAASi modestly preserve LVEF during anthracycline therapy. However, these benefits have not translated into significant reductions in overt heart failure or mortality, highlighting the gap between surrogate endpoints and clinically meaningful outcomes (37–40).

Beyond anthracyclines, exploratory data suggest a potential role of RAASi in other contexts. Observational studies have reported lower rates of dysfunction with trastuzumab (Moey 2019) and improved outcomes with immune checkpoint inhibitors (Chiang 2023). Perioperative RAASi exposure has also been linked with reduced long-term mortality after oncologic surgery (Oh 2022) (41–43).

Although various studies have demonstrated cardioprotective benefits from all three classes of RAASi—ACEi, ARBs, and MRAs—some network meta-analyses have aimed to directly compare their relative efficacy to identify the most effective agents for preventing chemotherapy-induced cardiotoxicity. Among these, several analyses consistently highlight enalapril and spironolactone as the most promising options. Ali Mir et al. and Xinye Li et al. found that spironolactone produced the greatest improvement in LVEF, followed by enalapril. Additionally, spironolactone significantly reduced troponin levels, while enalapril was associated with the largest drop in BNP and the lowest risk of clinical heart failure (44, 45).

The role of angiotensin receptor-neprilysin inhibitors (ARNI) in preventing CTR-CD in an emerging area of interest, particularly for patients receiving anthracyclines. Preclinical studies in animal models have shown that ARNI (sacubitril/valsartan) can prevent anthracycline-induced myocardial dysfunction by reducing oxidative stress, mitochondrial dysfunction and inflammatory response and these effects were more pronounced than with ARBs o ACEi alone (46, 47). Human data, however, remain limited (48). The ongoing PRADAII trial is specifically evaluating ARNI for prevention of cardiac dysfunction in breast cancer patients receiving chemotherapy (49).

Current official recommendations, as outlined in the 2022 European cardio-oncology guidelines (13), support the use of RAAS inhibitors for primary prevention of cardiotoxicity with a class IIa indication. This recommendation specifically applies to patients at high or very high risk of developing cardiac dysfunction who are undergoing treatment with anthracyclines, anti-HER2 agents, or other potentially cardiotoxic therapies. While the ESC guidelines represent the most comprehensive and detailed framework, recommendations from other major societies provide complementary perspectives that increase global relevance.

The ASCO clinical practice guidelines (2017) primarily addresses long-term survivorship, emphasizing baseline cardiovascular risk assessment and periodic monitoring with troponin and echocardiography in high-risk patients. Preventive pharmacological therapy is mentioned, but without strong class recommendations, reflecting the limited evidence base at that time (12). The AHA scientific statements (2019–2023) integrate cardio-oncology into broader heart failure prevention strategies, highlighting aggressive control of traditional cardiovascular risk factors. Pharmacological prophylaxis with RAAS inhibitors or beta-blockers is described as reasonable in high-risk patients, but without universal endorsement (50–52). The ESMO consensus recommendations (2020), on the other hand, adopt a more oncology-driven perspectives, stressing multidisciplinary collaboration and continuity of cancer treatment. While acknowledging the potential role of ACE inhibitors and beta-blockers, ESMO does not provide formal class of recommendations, instead focusing on structured monitoring multidisciplinary strategies (10). Taken together, these guidelines converge on the importance of baseline cardiovascular risk stratification, close surveillance of high-risk patients and multidisciplinary management, but diverge in the strength of recommendations for prophylactic pharmacotherapy. The ESC uniquely provides formal class IIa recommendations for RAASi and beta-blockers in high and very-high risk patients, whereas ASCO and AHA remain more cautious, and ESMO prioritize monitoring and oncological treatment continuity.

This heterogeneity underscores the need for harmonized global guidance and highlights the importance of ongoing larger and better-powered randomized trials in higher-risk populations, which are essential to refine the role of pharmacological prevention in cardio-oncology.

### 3.2 Beta-blockers

Although the use of beta-blockers (BBs) to prevent anthracycline-induced cardiotoxicity has been widely investigated, the evidence remains heterogeneous, with conflicting results across trials. An editorial contextualized this variability, noting that studies had important design limitations, and highlighted the CECCY trial as the largest placebo-controlled RCT evaluating carvedilol for primary prevention (53, 54). In CECCY, carvedilol did not reduce the incidence of LVEF decline ≥10%, but was associated with significantly lower troponin I levels and reduced diastolic dysfunction, suggesting subclinical benefit. This is corroborated by a recent meta-analysis by Attar et al., which included 17 RCTs (*n* = 1,291), and found that BBs attenuated the decline in LVEF after chemotherapy, although this did not translate into a statistically significant reduction in the incidence of cancer therapy–related cardiac dysfunction (55). Among included trials, CECCY was the largest and showed only modest clinical benefits. A troponin-guided strategy was further evaluated using carvedilol combined with candesartan in high-risk patients but found no significant protection against LVEF decline (56).

Recently, the rationale and design of the ongoing CARDIOTOX trial reinforce the need for large-scale studies powered for clinical outcomes. With over 1,000 planned participants, CARDIOTOX aims to clarify whether carvedilol prevents meaningful cardiovascular events, including heart failure, arrhythmias, and LVEF decline in anthracycline-treated patients (57).

### 3.3 SGLT2 inhibitors

Sodium-glucose co-transporter 2 inhibitors (SGLT2i) are a well-established class of drugs initially developed for glycemic control in diabetes, but their clinical use has expanded remarkably following the impressive evidence demonstrating benefits in patients with heart failure. However, their role in the prevention of cancer therapy-related cardiac dysfunction remains less defined and is currently under investigation in recent studies. As reviewed by Dhabour et al., there is strong biological plausibility that SGLT2i may counteract anthracycline-induced cardiotoxicity by attenuating oxidative stress, inflammation, and fibrosis, mechanisms shared with conventional heart failure pathology (58, 59).

A large retrospective analysis of over 17,000 propensity-matched diabetic patients undergoing potentially cardiotoxic chemotherapy was recently conducted (60). Interestingly, SGLT2i use was independently associated with a significant reduction in CTR**-**CD, heart failure events, and all-cause mortality. The EMPACARD-PILOT trial prospectively tested empagliflozin in high-risk breast cancer patients receiving anthracyclines (61). Compared to controls, those treated with empagliflozin had a markedly lower incidence of CTR**-**CD (6.5% vs. 35.5%, *p* = 0.005), with preserved LVEF and GLS at follow-up, despite no significant difference in NT-proBNP or troponin elevation. These findings, though promising, call for larger randomized trials to confirm the cardioprotective effect of SGLT2i in this setting.

In conclusion, SGLT2i appears to be safe and may reduce cardiovascular events in patient with cancer treated with anthracycline (62–65).

### 3.4 Other cardioprotective agents

A robust body of evidence, including meta-analyses and clinical trials, supports the use of statins for the primary prevention of cardiovascular disease (CVD), with a generally favorable balance of efficacy and safety. Meta-analyses consistently show that statins significantly reduce the risk of major cardiovascular events and CVD disease mortality in primary prevention population (66–68). Additionally, statins are associated with a small increased risk of adverse effects such as self-reported muscle symptoms, liver dysfunction and renal insufficiency, but not with clinically confirmed muscle disorders or diabetes (69).

Statins are generally well-tolerated in the oncology setting, with no significant increase in adverse events reported. Their cardioprotective effects are attributed to pleiotropic actions: reducing oxidative stress, inflammation, fibrosis and endothelial dysfunction—all mechanisms central to cancer-therapy induced cardiotoxicity (70, 71).

Multiple recent meta-analyses and RCTs demonstrate that statin therapy is associated with a significantly lower risk of CTR-CD and heart failure in cancer patients, reducing the incidence of anthracycline-induced CTR-CD by about 50%, with a smaller decline in LVEF compared to controls, a lower risk of heart failure and effective also in the trastuzumab-only population (72–74). However, some RCT found no significant difference in LVEF decline or CTR-CD incidence, highlighting, once again, heterogeneity in results and the need for further large-scale trials (73).

Metformin, a first-line antidiabetic agent, has attracted attention in cardio-oncology because of its potential pleiotropic cardioprotective effects beyond glycemic control. Mechanistically, metformin activates AMP-activated protein kinase (AMPK), which reduces oxidative stress, improves mitochondrial function and attenuates apoptosis. These pathways overlap with those implicated in anthracycline-induced cardiotoxicity, providing a strong biological rationale for its use (75, 76).

Clinical evidence, however, is limited and influenced by study design, cancer type and patient population. Observational studies in diabetic cancer patients have reported lower rates of cardiotoxicity, heart failure events and mortality in those receiving metformin compared with non-users. Some retrospective analyses also suggest improved cancer-related outcomes, likely due to metformin's anti-proliferative and insulin-sensitizing properties. However, prospective randomized controlled trials specifically designed to assess metformin's cardioprotective effect in non-diabetic cancer patients are lacking (75, 77–79).

From a guideline's perspective, ESC and ESMO mention metformin as a promising but experimental option, without formal class of recommendation grading. In summary, metformin is biologically plausible and supported by encouraging preclinical and observational evidence, but its role in routine cardioprotection remain investigational. Ongoing and future prospective trials are essential to clarify whether metformin should be considered beyond diabetic population, potentially as a repurposed therapy for anthracycline-induced cardiotoxicity (10, 13).

Dexrazoxane is currently the only FDA-approved drug for the prevention of anthracycline-induced cardiotoxicity. Its mechanism of action is primarily related to iron chelation, which reduces the formation of anthracycline-iron complexes responsible for oxidative stress and free radical-mediated myocardial injury. In addition, dexrazoxane interferes with topoisomerease IIBeta inhibition in cardiomyocytes, thereby mitigating DNA damage and cell death. Clinical evidence of its use is robust, especially in pediatric oncology. Multiple randomized trials have demonstrated that dexrazoxane significantly reduces the incidence of left ventricular dysfunction, troponin elevation and the risk of heart failure, though concerns about potential interference with chemotherapy efficacy and the risk of secondary malignancies initially limited its widespread adoption. More recent analyses, however, have not substantiated these concerns, and international consensus now supports its safe use in carefully selected populations (80–83).

The ESC cardio-oncology guidelines endorse dexrazoxane as a class IIa recommendation in patients expected to receive high cumulative dose of anthracyclines (> 250–300 mg/mq of doxorubicin equivalent) or those at very high-risk of cardiotoxicity. Similarly, the ASCO guidelines acknowledge its cardioprotective role but highlight the need to balance benefit with oncologic efficacy. Moreover, the ESMO consensus recommends its use in patients requiring high-dose anthracyclines when alternative regimens are not feasibile (10, 12, 13).

## 4 Non-pharmacological strategies

Non-pharmacological strategies represent an essential complement to pharmacological cardioprotection in cancer patients. These strategies include behavioural interventions, lifestyle modifications, psychological support, and a multidisciplinary approach aimed at improving quality of life and reducing overall cardiovascular risk.

Unlike drug-based approaches, the supporting evidence for lifestyle-oriented strategies—such as exercise and nutrition—is largely driven by improvements in functional parameters and quality of life, rather than by robust reductions in hard clinical endpoints such as heart failure or mortality. Most available trials are small, heterogeneous and rely on surrogate outcomes (e.g., LVEF, GLS, VO2 peak or peak oxygen consumption), which limits the certainty of evidence. An overview of non-pharmacological approaches to prevent cancer therapy-related cardiac dysfunction is shown in Figure 1.

Nevertheless, both the ESC 2022 cardio-oncology guidelines and consensus documents from ASCO, AHA and ESMO emphasize the importance of integrating exercise and nutritional support into survivorship care (10, 12, 13). These strategies therefore play a pivotal role in multidisciplinary cardio-oncology, even if their formal class-of-recommendation grading is less strong than for pharmacological agents such as RAASi or dexrazoxane.

### 4.1 Cardiovascular risk assessment

Accurate cardiovascular risk assessment through a detailed medical history, physical examination and risk stratification tools is the cornerstone of cardio-oncology, enabling tailored surveillance and prevention strategies.

Among these tools, the Heart Failure Association–International Cardio-Oncology Society (HFA-ICOS) score is currently the most widely adopted and validated. It integrates patient-related factors (age, comorbidities), therapy-related risk (type and dose of anticancer treatments) and cardiac biomarkers, stratifying patients into low, moderate, high and very high-risk categories. The Heart Failure Association–International Cardio-Oncology Society (HFA-ICOS) score is summarized in Table 2. Prospective studies have shown that HFA-ICOS predicts the incidence of anthracycline-induced cardiotoxocity and guides follow-up intensity (13). Timely use of this score is crucial for planning both pharmacological and non-pharmacological preventive strategies (84). However, its derivation mainly from European cohorts may limit generalizability to non-European populations, pediatric settings and patients receiving newer agents such as immune checkpoint inhibitors.

Other models, including SCORE2 or AH-HA, have been explored but lack formal validation in oncology cohorts. Overall, the robustness of available score is moderate: they provide a practical framework for clinical use yet rely on surrogate endpoints and observational validation rather than randomized evidence (85–88).

### 4.2 Control of cardiovascular risk factors

Aggressive management of modifiable cardiovascular risk factors (hypertension, obesity, dyslipidemia, diabetes, and smoking) is a low-cost, high-yield strategy to reduce CTR-CD. Numerous studies have demonstrated that the presence of these factors significantly increases the risk of developing cardiac damage during cancer therapies (89–94). Addressing these factors remains a primary preventive strategy.

Contemporary guidelines strongly emphasize this approach. The 2024 ESC hypertension guidelines recommend a blood pressure target of SBP 120–129 mmHg and DBP 70–79 mmHg if tolerated (otherwise ALARA or as low as reasonably achievable) in high-risk patients with clinical hypertension, including those with cancer. The 2019 ESC/EAS lipid guidelines advise initiating statins in patients with a 10-yeas ASCVD risk ≥7.5% and observational data suggest that statins attenuate CTR-CD. Similarly, AHA scientific statement and the ASCO guidelines endorse aggressive control of risk factors as a primary prevention strategy (12, 50, 52, 95, 96).

Despite the strong consensus, real-world adherence remains suboptimal. Registry data show that hypertension and dyslipidemia are often undertreated in cancer patients, leading to preventable cardiovascular complications (97, 98). Incorporating structured cardio-oncology rehabilitation and early referral to cardiology could bridge this gap.

Evidence quality for risk factor control is high for overall cardiovascular benefit, but indirect for CTR-CD prevention, highlighting the need for oncology-specific implementation studies.

### 4.3 Physical activity and rehabilitation

Exercise is one of the most consistently recommended non-pharmacological strategies. Randomized controlled trials, including the ONCORE trial, and several meta-analyses suggest that aerobic and combined aerobic-resistance training modestly improve LVEF, GLS, VO2 peak and diastolic function in patient receiving anthracyclines. However, sample sizes are small, follow-up is short and clinical outcomes such as heart failure hospitalization or survival are rarely captured (99, 100).

A recent study by our group (101) showed that regular physical activity is associated with reduced cardiovascular and overall mortality, improved cardiorespiratory fitness, and decreased symptoms of chemotherapy-induced cardiotoxicity. Exercise also promotes favorable cardiac remodeling, improves endothelial function, and reduces oxidative stress, contributing to better quality of life in cancer patients. A schematic overview of different types of physical activity used in cardio-oncology is shown in Table 3.

Systematic reviews grade the certainty of evidence as low-to-moderate for surrogate endpoints and for low for clinical outcomes. Heterogeneity in exercise prescription (intensity, timing, delivery) further limits generalizability. Nevertheless, safety is consistently demonstrated, and adherence is feasible with supervised programs (100, 102).

Guidelines reflect these nuances: the ESC provide a classe I recommendation for exercise in cancer survivors as part of cardiovascular prevention, while AHA and ASCO statements support supervised exercise but without strong class grading for CTR-CD prevention. Future priorities include large multicenter RCTs with standardized exercise protocols, integration into cardiac rehabilitation frameworks and evaluation of long-term outcomes beyond functional parameters (10, 12, 13).

In summary, exercise is safe and beneficial for functional endpoins and quality of life, but robust evidence for hard outcomes is still lacking.

### 4.4 Nutrition and nutritional support

Nutrition interventions are essential for holistic cardioprotection, though direct evidence for CTR-CD prevention remains limited. Preclinical studies and small human trials suggest that adherence to a Mediterranean diet and supplementation with antioxidants (e.g., coenzyme Q10, zinc, selenium, polyphenols) may attenuate anthracycline-induced oxidative stress and reduce biomarker release (103–105). However, results are inconsistent, and the overall certainty of evidence is very low for specific supplements (103).

In contrast, the role of nutritional screening and support is supported by more solid evidence. Malnutrition and sarcopenia predict poor treatment tolerance, increased toxicity and higher cardiovascular risk in both pediatric and adult oncology populations (106, 107). Early involvement of dietitians and individualized support has been shown to improve outcomes and quality of life, with moderate evidence for clinical benefit (108, 109).

Guidelines from ESC and ESMO recommend systematic malnutrition screening and personalized nutritional counseling but stop short of endorsing any particular dietary supplement for CTR-CD prevention. ASCO and AHA also emphasize weight control, diabetes prevention and general cardiometabolic health as key targets during survivorship (10, 12, 13).

In summary, nutrition is indispensable for global cardiovascular and oncologic outcomes, but its role in specific CTR-CD prevention remains exploratory. Stronger evidence supports structured nutritional assessment and support rather than isolated supplement use.

### 4.5 Psychological support and mind-body techniques

Psychological support and stress management strategies (such as yoga, mindfulness, and meditation) have shown promise in improving the quality of life and psychological well-being of cancer patients. Recent studies indicate that meditation, when incorporated into cardiac rehabilitation programmes, reduces anxiety, depression, and stress in patients with coronary artery disease compared with standard care (110–113).

In oncology, particularly among women with breast cancer, mindfulness and loving-kindness practices have been shown to alleviate pain, fatigue, and anxiety, with potential benefits for heart rate modulation (114, 115).

Couple-based meditation interventions, including online programmes, have demonstrated positive effects on quality of life and symptom management for both cancer patients and their partners (116).

A recent randomized study found that regular Buddhist walking meditation may mitigate anthracycline-related cardiotoxicity, improving vascular function and quality of life compared with controls (117).

However, robust evidence of a direct effect of psychological support on cardiotoxicity prevention is currently lacking (118). Nevertheless, psychological support is recommended as an integral part of a multidisciplinary approach, facilitating adherence to follow-up, symptom management, and emotional processing, with potential indirect cardiovascular benefits.

### 4.6 Multidisciplinary approach

Continuous communication between cardiologists and oncologists is essential for managing cardiovascular risk in cancer patients. This integrated approach enables personalized prevention, monitoring, and treatment strategies, ultimately improving patient prognosis (119–121).

Balancing the benefits of cancer therapies against cardiovascular risks and promoting joint education between cardiologists and oncologists are fundamental (122–124).

The creation of dedicated cardio-oncology units is recommended to ensure comprehensive and coordinated care for cancer patients, bridging existing gaps in clinical practice (125, 126).

## 5 Future prospectives

Current research in the field of cardio-oncology prevention is increasingly focused on developing innovative strategies to enhance diagnostic accuracy and enable earlier identification of patients at risk of cancer therapy–related cardiac dysfunction (CTR-CD). These approaches aim to detect subclinical cardiac damage and to stratify individual risk in a more personalized way, with the ultimate goal of integrating precision medicine more effectively into routine clinical practice.

To further strengthen the clinical applicability of these approaches, there is a growing need for well-designed comparative clinical trials aimed not only at evaluating the efficacy of pharmacological and non-pharmacological preventive strategies, but also at determining which of these approaches—or what combination thereof—offers the most effective and sustainable protection in specific patient populations.

### 5.1 Medical genetics

A field of growing interest is pharmacogenomics and the study of genetic polymorphisms. Several studies have identified genetic variants, such as single nucleotide polymorphisms (SNPs), that increase individual susceptibility to the cardiotoxic effects of cancer therapies, particularly anthracyclines and HER2 inhibitors. For example, polymorphisms in the CYBA, RAC2, CYP3A5, ABCC1, ABCC2, and HER2 genes have been associated with an increased risk of cardiac toxicity. Integrating pharmacogenomics into clinical practice could allow for a more precise individual risk stratification compared to traditional risk factors, enabling the identification of more susceptible patients and the implementation of personalized prevention strategies, such as closer cardiological monitoring or early use of cardioprotective drugs. The use of genetic testing and polygenic risk scores represents a promising frontier in cardio-oncology, with the potential to make cancer therapies safer and more targeted. However, the clinical validation of these genetic markers is still ongoing, and further studies are needed to define their actual impact on the prevention of CTR-CD (127–134).

Medical genetics therefore offers promising tools for personalized prevention of cardiotoxicity, but their routine application—considering costs and turnaround times—requires careful patient selection to identify those who could truly benefit (135–139).

### 5.2 Omics sciences

Another emerging tool is represented by omics sciences, particularly metabolomics, which can identify early biomarkers of cardiotoxicity induced by both chemotherapy and radiotherapy. Recent studies have demonstrated that the analysis of plasma metabolic profiles can detect alterations associated with cardiac damage before the clinical onset of symptoms. In particular, in thoracic radiotherapy, changes in steroid hormone and vitamin E metabolism have been linked to an increased risk of cardiotoxicity (140–142).

*In vitro*, exposure to common chemotherapeutic agents (such as anthracyclines or 5-fluorouracil) has shown metabolic changes, with an increase in metabolites associated with inflammation and oxidative stress (143, 144).

Furthermore, a clinical study conducted by the Cleveland Clinic on patients treated with chemotherapy identified 13 plasma proteins and 14 metabolites associated with the development of left ventricular dysfunction assessed via echocardiography (145).

The integrated omics approach (genomics, proteomics, transcriptomics, and metabolomics) offers new opportunities for the early identification of biomarkers and for understanding the molecular mechanisms underlying cardiotoxicity, overcoming the limitations of traditional markers that only change after significant damage has occurred (146, 147).

Metabolic profiling enables a more sensitive and earlier characterization of cardiac injury phenotypes, supporting precision medicine strategies for surveillance and prevention (148).

However, research in this field is still under development, and further studies are needed to validate and standardize the metabolite panels to be used in clinical practice (146, 148).

### 5.3 Telemedicine

Telemedicine is emerging as a promising tool for the prevention and management of cardiovascular toxicity in cancer patients. Recent studies suggest that telemonitoring programs, such as the ON-CARDIO model, can facilitate the early detection of cardiac complications related to cancer therapies, especially in certain patient groups, such as those with colorectal cancer, although the reasons for this selection need further clarification. This model involves continuous telemonitoring of parameters such as ECG, blood pressure, and biomarkers, aiming for the timely detection of arrhythmias and cardiac dysfunction during therapy (149).

American and European guidelines emphasize the importance of a multidisciplinary and personalized approach to the cancer patient, which also includes the use of digital technologies for continuous surveillance (9, 10, 118, 150).

A recent systematic review has shown that telemedicine, particularly when integrated with remote monitoring and specialist consultations, can reduce mortality and hospitalizations for cardiovascular causes in patients with heart failure, suggesting potential benefits in cardio-oncology as well (151).

However, specific research on the effectiveness of telemedicine in preventing cardiovascular toxicity in cancer patients is still in its early stages, and further studies are needed to confirm these results (152).

### 5.4 Artificial intelligence in cardio-oncology

Artificial intelligence (AI), thanks to its ability to integrate and analyze large amounts of clinical, instrumental, and imaging data, is revolutionizing the prevention of cardiotoxicity in cancer patients, providing advanced tools for risk stratification, early diagnosis, and monitoring of complications related to cancer therapies. Machine learning and deep learning models, such as neural networks and random forest algorithms, applied to clinical data, echocardiographic parameters, and electrocardiograms (ECGs), have shown good predictive capability for the risk of cardiac dysfunction and heart failure in patients treated with anthracyclines and trastuzumab, allowing for early identification of at-risk individuals and potentially guiding personalized prevention strategies (153–158).

The application of AI to ECG analysis can detect subclinical alterations of left ventricular function with diagnostic performance comparable to echocardiography, enabling risk stratification even before the initiation of therapy (155–157). In addition, two recent large-scale, retrospective cohort studies applied validated AI models to ECG to predict and stratify the risk of CTR-CD in cancer patients receiving anthracyclines or trastuzumab (157, 159). While these are not randomized clinical trials, they represent robust, real-world clinical evidence supporting AI's role in CTR-CD prevention and early detection.

Moreover, integrating AI with advanced imaging data (echocardiography, cardiac magnetic resonance, PET) and multi-omics biomarkers allows for an even more precise and personalized cardiovascular risk assessment, facilitating early diagnosis and monitoring of complications (160–164).

However, these tools must be validated and standardized in multicenter studies and integrated into routine clinical practice to ensure equitable access and reliable results. Additionally, it is essential to adequately train healthcare professionals in the use of these technologies (165–167).

A 2024 systematic review and meta-analysis evaluated the efficacy of AI in automating cardiothoracic ratio measurement on chest x-ray, which is relevant for screening and monitoring cardiac dysfunction, including CTR-CD. This meta-analysis included 14 studies with over 70,000 images, demonstrating that AI models are highly accured (pooled AUC 0.959) and efficient compared to manual methods (168).

There are a high-quality meta-analysis and multiple large-scale clinical studies supporting the use of AI for the prevention and detection of CTR-CD, though randomized clinical trials are still lacking.

## 6 Critical appraisal and evidence gaps

Despite the growing body of literature on cardiotoxicity prevention, several limitations temper the strength of current evidence. First, most pharmacological trials are relatively small, single-center studies with short follow-up, often relying on surrogate endpoints such as LVEF or GLS rather than hard outcomes like heart failure, hospitalization or mortality. Even meta-analyses, though more robust, are influenced by heterogeneity in study design, patient populations and definitions of cardiotoxicity. As a result, while agents such as RAAS inhibitors, beta-blockers, statins and dexrazoxane demonstrate consistent preservation of LVEF, their impact on long-term clinical outcomes remains uncertain.

For non-pharmacological interventions, evidence quality is further constrained. Exercise and nutrition strategies are supported by safety data and improvements in functional parameters, but most trials remain underpowered, heterogeneous in protocols, and focused on surrogate markers. This limits generalizability and prevents strong class-of-recommendation grading in guidelines.

Emerging tools such as artificial intelligence, telemedicine and omics sciences show considerable promise, yet are still in exploratory phases. Most studies are retrospective or based on pilot cohorts, underscoring the urgent need for multicentered, prospective validation before widespread clinical adoption.

Finally, while current guidelines converge on the importance of cardiovascular risk factor management, they diverge in the strength of recommendations for prophylactic pharmacotherapy. This reflects a broader gap in harmonized, global consensus and highlights the need for large-scale, international randomized trials with longer follow-up.

In summary, the field is rapidly evolving but still characterized by significant evidence gaps. Addressing these limitations will be essential to move from surrogate-based preventive strategies to interventions that meaningfully improve survival and quality of life in cancer patients.

## 7 Conclusions

Cardiotoxicity remain a central challenge in modern oncology, significantly affecting prognosis and survivorship. Strongest evidence supports dexrazoxane, RAASi and beta-blockers in selected high-risk patients, as well as strict control of cardiovascular risk factors, which together represent the cornerstone of current preventive strategies. Exercise and nutritional interventions are safe and improve functional capacity and quality of life, although their impact on hard cardiovascular outcomes is less well established.

Promising approaches—including statin, metformin, SGLT2i and emerging technologies such as AI, telemedicine and omics sciences—expand the preventive armamentarium but remain investigational.

International guidelines converge on baseline risk stratification and multidisciplinary management yet diverge in the strength of recommendation for prophylactic pharmacotherapy. This underscores the urgent need for harmonized global guidance supported by multicenter randomized trials with longer follow-up, focusing on clinically meaningful endpoints.

In conclusion, cardio-oncology prevention must evolve towards an integrated, evidence-based and globally applicable model, combining validated pharmacological therapies, lifestyle interventions and innovative technologies to optimize survival and quality of life for cancer patients and survivors.
